# Antimicrobial and Anti-Biofilm Activities of Coffea arabica L. Against the Clinical Strains Isolated From Diabetic Foot Ulcers

**DOI:** 10.7759/cureus.52539

**Published:** 2024-01-19

**Authors:** Mohammad Zubair

**Affiliations:** 1 Medical Microbiology, University of Tabuk, Tabuk, SAU

**Keywords:** inhibition type, mrsa, esbl, biofilm inhibition, coffee extract, diabetic foot ulcer

## Abstract

Diabetes-related complications such as diabetic foot infections foster resilient biofilms, complicating treatment. Innovative therapeutic solutions are urgently needed to address this challenge. In this research, coffee bean powder (green coffee been powder [GCBP], roasted coffee bean powder [RCBP], and spent coffee powder ground [SCPG]) was extracted and assessed for its ability to impede biofilm formation and associated functions in extended-spectrum beta-lactamase (ESBL) and methicillin-resistant *Staphylococcus aureus* (MRSA)-positive biofilm-forming strains of *Pseudomonas aeruginosa (P. aeruginosa)*, *Escherichia coli (E. coli)*, and *Staphylococcus aureus (S. aureus) *obtained from foot ulcers.

GCBP exhibited notable effectiveness in reducing biofilm formation, ranging from 17-76% in monocultures and 17-66% in mixed cultures. It significantly disrupted motility in *P. aeruginosa* and *E. coli*, a crucial factor influencing biofilm establishment. The critical biofilm-related functions for attachment and maintenance such as cell surface hydrophobicity and exopolysaccharide production were significantly inhibited at sub-MICs. Notably, GCBP elicited statistically significant reductions (29-59% in monocultures and 28-45% in mixed cultures) in pre-formed biofilms. The reduction in bacterial chitinase activity upon exposure to GCBP implies a potential mechanism for its ability to inhibit biofilm formation. This study emphasizes the potential of green coffee bean extract in tackling antibiotic-resistant bacterial biofilms associated with diabetic foot ulcers, suggesting innovative strategies for infection management through mechanistic understanding and optimized applications.

## Introduction

In recent years, coffee has risen beyond its role as a beloved beverage to become a subject of considerable scientific interest, particularly in the Middle East, where its per capita consumption is notably high. Beyond its popular taste and cultural significance, emerging research underscores the potential health benefits associated with incorporating natural products, like coffee extracts, into our daily diets for disease prevention. This growing interest stems from the notion that integrating such natural compounds might offer enhanced safety and efficacy compared to conventional medications, as evidenced in the study by Akhlaghi et al. [1].

Recognized for its stimulant effect primarily due to caffeine, coffee also encompasses an array of compounds like carbohydrates, lipids, nitrogenous elements, vitamins, minerals, alkaloids, and phenolic compounds, contributing to its diverse properties [2, 3]. However, the coffee industry generates substantial by-products annually, prompting proposals for their value addition in producing mushrooms, enzymes, organic acids, biofuels, and fertilizers [4]. Approximately 6 million tons of spent coffee powder ground (SCPG) are generated worldwide during instant coffee production and brewing, comprising an oil fraction, crude fiber, and various bioactive compounds like caffeine, trigonelline, phenolics, minerals, lignin, and melanoidins, varying based on coffee bean type and roasting conditions [5]. Green coffee bean powder (GCBP), roasted coffee bean powder (RCBP), and SCPG exhibit antioxidant, antiproliferative, and antibacterial effects. Studies by Daglia et al. [6] demonstrated antibacterial properties in RCBP, while Monente et al. [7] indicated antimicrobial activities of RCBP and SCPG against Gram-positive bacteria and yeast. DFUs are a common and often debilitating complication arising from diabetes. These ulcers frequently become infected, transforming into chronic wounds. Alarmingly, a significant proportion of these ulcers are affected by bacterial biofilms, presenting a formidable obstacle in wound management due to their inherent resistance to conventional antibiotics and the host immune response. As elucidated by Malik et al. [8], this resistance contributes to prolonged healing times and increases the risk of complications, exacerbating the challenges in treating such wounds. The research focused on the antimicrobial properties of coffee extracts has gained substantial traction due to the uncontrolled prevalence of bacterial resistance to conventional medications, as substantiated by Stefani et al. [9]. Additional studies conducted outside the Middle East [7, 10-12] further bolster these findings, illustrating similar results in minimum inhibitory concentrations against various bacterial strains. Based on the facts related to coffee powder, this research aims to conduct an in-depth study on the antibacterial and antibiofilm effects of coffee extracts against bacterial strain isolates from DFU/DFI as well as to understand the basic mechanism of these extracts' antibiotic and anti-biofilm activity at minimal inhibitory concentrations (MIC) and sub-MIC levels, in mono and mixed cultures of three most prevalent bacterial infections associated with DFUs: *Pseudomonas aeruginosa (P. aeruginosa), Escherichia coli (E. coli), *and* Staphylococcus aureus (S. aureus).*

## Materials and methods

Collection of samples

Green Arabian coffee beans (*Coffea arabica* L.), was collected from the local market in Tabuk, Kingdom of Saudi Arabia. 

Preparation of coffee sample extract

For this study, 50 g of unroasted green coffee seeds underwent a roasting process in a coffee roasting machine set at a temperature of 190± 5 ◦C for a duration of 15 minutes. Following this, the roasted seeds were ground using a coffee grinder. A portion of 10 g was percolated through a coffee filter, allowing 500 mL of water to pass through via a coffee brewing machine. The remaining spent coffee grounds (SCG) were subsequently dried at 60 °C for 5-6 hours and then stored at -20 °C. To create the aqueous extracts, a mixture consisting of 5 g of GCBP, 5 g of RCBP, and 5 g of SCPG was combined with 50 mL of water and sonicated for one hour at 45 °C. After centrifugation for 30 minutes at 1800 rpm, the upper layer was removed, and the process was repeated after adding an additional 50 mL of water. The resulting supernatants were combined, filtered, and air-dried in a sterile chamber. The yield was calculated using the formula: yield = (grams of extracts obtained × 100 / grams of macerated coffee beans). These extracts were then stored at -20 °C for future use [13].

Collection of bacterial samples and measurement of antibiotic sensitivity

A characterized 45 (15 *E. coli* + 15 *S. aureus *+ 15 *P. aeruginosa*) strains were collected from the hospital. All strains were isolated from diabetic patients having ulcers in their feet. Kirby Bauer disk diffusion method was used for the susceptibility test as per Clinical and Laboratory Standards Institute (CLSI) [14] guidelines on Mueller-Hinton agar. Amikacin (30µg), ceftazidime (30µg), cefepime (30 μg), levofloxacin (5 μg), tobramycin (10µg), piperacillin (100 μg), imipenem (10 μg), cefoperazone (75 μg), cefoperazone/sulbactam (75/10 μg), cefotaxime (30µg), cefotaxime/clavulanic acid (30/10µg), piperacillin/tazobactam (100/10 μg), cefepime clavulanic acid (30/10µg), sparfloxacin (5 μg), tobramycin (10 μg), erythromycin (15 μg), gentamicin (10 μg), oxacillin (1 μg), ciprofloxacin (5µg), cefoxitin (30 μg), and vancomycin (30 μg) were used in this study (Hi-Media labs, Mumbai, India). Interpretation of result as suggested by the manufacturer's recommendation (Hi-Media labs, Mumbai, India).

Extended-spectrum beta-lactamase (ESBL) and methicillin-resistant Staphylococcus aureus (MRSA) test

ESBL producers were detected by testing the sensitivity of ceftazidime and cefotaxime alone and in combination with clavulanic acid (10 mg), as recommended by the CLSI [15]. For MRSA detection, a sterile swab was dipped in the *S. aureus* suspension (0.5 McFarland) and plated onto Mueller Hinton agar (MHA). Oxacillin discs (1 μg) were placed onto the surface of inoculated agar and plates were incubated overnight at 30°C. An isolate was classified as resistant to oxacillin when the inhibition zone was ≤14 mm in diameter [16]. The control strains used in this study were *E. coli *ATCC 25922 (non-ESBL-producer), *Klebsiella pneumoniae* 700603 (ESBL-producer), and *S. aureus* (ATCC 25923).

Biofilm formation in 96-well microtiter plates

Biofilm formation was examined by the quantitative method in 96-well flat bottom plates. For each clinical strain, biofilm assays were performed in triplicate and the mean biofilm absorbance value was determined. Biofilm formed were classified as weak (OD590 0.1 to ≤0.400), moderate (OD590 > 0.400), and strong (OD590 > 0.800) according to the method described elsewhere [8, 16]. 

Minimum inhibitory concentration (MIC) activity of GCBP, RCBP, and SCPG extracts

MIC of coffee extract of GCBP, RCBP, and SCPG against drug-resistant, biofilm-forming strains of *P. aeruginosa*, *E. coli,* and *S. aureus *was determined using the standard micro-broth dilution method of CLSI [17]. The lowest concentration at which there was no measurable OD after 18 hours of incubation at 37°C at 600nm was considered for further experiments with slight modification [18].

Effect of sub-MICs of GCBP, RCBP, and SCPG extract on the viability of test bacteria

To assess the effect of sub-MICs of GCBP, RCBP, and SCPG extract on the viability of test bacteria, a growth curve analysis was performed. Briefly, cells were inoculated into 100 ml Mueller Hilton broth (MHB) and cultivated in the presence or absence of sub-MICs of GCBP, RCBP, and SCPG extract in triplicates. The culture set-up was incubated at 37 °C and the OD was monitored at two-hour intervals for up to 18 hours at 600 nm.

Effect of GCBP, RCBP, and SCPG extracts on the biofilms of mono and mixed species

In the inhibition tests, microtitre plate-inoculated bacteria were treated with 1/18-1/2 times MICs of GCBP, RCBP, and SCPG extract, and the mixture was then incubated for 48 hours at 37 °C. Biofilm inhibition was quantified using the methods outlined in the preceding section. The estimation of mixed biofilm production followed Zhang et al.'s instructions [19]. To put it briefly, each strain (*P. aeruginosa + E. coli, S. aureus + P. aeruginosa, *and *E. coli + P. aeruginosa*) was cultured in Tryptic soy broth (TSB) overnight at 37°C and then diluted to 1 x 106 CFU/ml in TSB. Each bacterium was mixed in equal quantities (1:1), and 100 µl of the combined bacterial suspension was applied to each well of the polystyrene 96-well tissue-culture plates. 100 µl of fresh TSB with varying concentrations of GCBP, RCBP, and SCPG extracts (1/16-1/2 times MIC) was added to each well. Positive controls were wells devoid of any additives, while negative control wells had solely TSB. Following a 48-hour incubation period at 37°C, the plates were gently cleaned with 1X phosphate-buffered saline (PBS; pH 7.4) and then dyed for 30 minutes at room temperature using 100 μl of 0.1% crystal violet (Sigma-Aldrich, St. Louis, MO). After solubilizing CV in 95% ethanol, excess crystal violet was eliminated by washing, and biofilm was measured by measuring the equivalent OD590 nm of the supernatant. 

Effect on exopolysaccharides (EPS) and swarming motility

To extract the EPS, the cell-free supernatant of test strains treated with GCBP (0.25-0.5 times MIC) and those that were not treated were collected, combined in a 3:1 ratio with cooled ethanol, and incubated at 4 °C for 16-18 hours [20]. The Dubois procedure was used to estimate the extracted EPS and the glucose standard curve was used to quantify it [21]. After adding 0.25-0.5 times MIC of GCBP extract, strains of *P. aeruginosa *and *E. coli *were point-injected into LB plates, which were then cultured for 24 hours at 37 °C. As a control, plates were not loaded with GCBP extract. To measure the impact on swarming motility, the diameter of the swarm on treated and untreated plates was measured.

Effect on cell surface hydrophobicity (CSH)

Microbial adhesion to hydrocarbons (MATH) assay was employed to determine the CSH of treated and untreated test strains [16]. Overnight-grown cultures of the test strains (1 ml) were added briefly to microcentrifuge tubes containing 100 μl xylene and sub-inhibitory concentrations of GCBP extracts. Control groups were not given any treatment. Vigorous vortexing of the samples was done for two minutes and then for separation of the two phases 10-min incubation at room temperature was given. The aqueous phase was read at 530 nm and percent hydrophobicity was determined using the formula: %hydrophobicity = ­1 − OD after vortexing ∕ OD before vortexing‑ × 10

Effect on the outer membrane disruption

An N-Phenyl-1-naphthylamine (NPN) absorption experiment was used to investigate the influence of the extracted coffee phytochemical on the biofilm-positive bacterial outer membrane permeability [22]. In brief, test strains were treated with 0.5x MIC and 0.25xMIC in a final volume of 1mL and incubated at 37°C for 1 hour. After that, the cell suspensions were rinsed and resuspended in 1mL of 0.5% NaCl. TCI (Japan) NPN solution in ethanol (100 mM) was added to 200 L of cells to achieve a final concentration of 0.75mM. At room temperature, background fluorescence was measured with an excitation wavelength of 350 nm and an emission wavelength of 420 nm for subtraction. NPN integrated into the membrane enhanced fluorescence when the outer membrane permeability increased due to the inclusion of the coffee phytochemical component. For the 100% maximum dye leakage release, Triton X-100 (0.1%; v/v) was utilized as a positive control.

The following equation was used to convert the values to % NPN uptake: % NPN uptake = (Fobs- F0)/(F100-F0)x100, where Fobs is the observed fluorescence at a specific compound concentration, F0 is the initial fluorescence of NPN with the cells in the absence of compound, and F100 is the fluorescence of NPN with the cells in the presence of compound. 

Effect on chitinase activity

Chitinase activity was estimated by fluorescence-based assay method (Sigma Aldrich USA), the sensitivity of the functional assay was 50.0 relative fluorescent unit (RFU)/ml with intra- and inter-assay CV % were 6.9 and 7.8 respectively.

Effect of GCBP on disruption of pre-formed biofilms

The pre-established biofilm of the test strains was cultivated for 24 hours in microtiter plates. After incubation, any cells that didn’t adhere were eliminated through a washing process. Fresh growth medium, with or without 0.5 times the MIC of GCBP, was introduced into each well and allowed to statically incubate at 37 ℃ for another 24 hours. Cells that remained unattached were washed away, and the adhering cells underwent staining with crystal violet. Subsequent measurements were taken at 585 nm, following the previously described method [16].

Statistical analysis

All experiments were performed in triplicates and the data obtained from experiments were presented as mean values; the difference between the control and test were analyzed using Student’s t-test on SigmaPlot v. 15 software (Systat Software, Inc., San Jose, CA). 

## Results

Drug‐resistance profiling and phenotypic ESBL and MRSA detection

*P. aeruginosa* (15 strains), *E. coli* (15 strains), and *S. aureus* (15 strains) were collected from hospitals in Tabuk city. The antibiotic resistance profile was estimated, and a double disk synergy test was used for the detection of ESBL among isolated *P. aeruginosa* and *E. coli.* Among 30 isolates, 10 (33%) were found to be ESBL-positive. Also, among *S. aureus*, 40% (n=6) were found to be MRSA (Table 1).

**Table 1:** Antibiotic resistance pattern of P. aeruginosa, E. coli, and S. aureus as well as the ESBL and MRSA patterns ESBL: extended-spectrum beta-lactamase, MRSA: methicillin-resistant *Staphylococcus aureus*

Parameters	n (%)
Resistance pattern of *P. aeruginosa* (n=15)	
Amikacin	9(60)
Ceftazidins	8(53)
Cefepime	8(53)
Levofloxacin	11(73)
Sparfloxacin	9(60)
Tobramycin	10(66)
Piperacillin	9(60)
Resistance pattern of *E. coli *(n=15)	
Amikacin	6(40)
Ceftazidins	7(46)
Cefepime	7(46)
Levofloxacin	12(80)
Sparfloxacin	9(60)
Tobramycin	12(80)
Piperacillin	6(40)
Resistance pattern of *S. aureus* (n=15)	
Amikacin	4(26)
Erythromycin	6(40)
Ciprofloxacin	8(53)
Gentamycin	9(60)
Levofloxacin	10(66)
Oxacillin	6(40)
Vancomycin	0(0)
ESBL pattern	
ESBL preliminary test for *P. aeruginosa*	
Ceftazidime	8(33)
Cefotaxime	9(60)
ESBL preliminary test for *E. coli*	
Ceftazidime	7(46)
Cefotaxime	8(53)
ESBL confirmatory test for *P. aeruginosa*	
Ceftazidime / ceftazidime clavulanuc acid	5(33)
Cefotaxime/ cefotaxime clavulanuc acid	6(40)
ESBL confirmatory test for* E. coli*	
Ceftazidime / veftazidime clavulanuc acid	5(33)
Cefotaxime/ cefotaxime clavulanuc acid	5(33)
MRSA results for* S. aureus*	
Oxacillin	6(40)

Biofilm formation among isolated *P. aeruginosa* (PA), *E. coli *(EC) and *S. aureus* (SA) strains

All bacterial strains underwent a biofilm assay using crystal violet staining, leading to their categorization into strong, intermediate, or weak biofilm formers. Among the tested strains, four strains of *P. aeruginosa*(namely, PS2, PS3, PS11, and PS14), three strains of *E. coli *(EC 1, EC8, and EC10), and three strains of *S. aureus* (SA3, SA8, and SA10) exhibited strong biofilm formation capability. Conversely, three strains of *P. aeruginosa*, five strains of *E. coli*, and three strains of *S. aureus* demonstrated a lack of biofilm formation. These findings are summarized in Table 2. Based on the robust biofilm-forming capacity, the following 3 strains in each genus [strong biofilm formers *P. aeruginosa *strains PS2, PS3, PS11, and PS14, the three *E. coli* strains (EC 1, EC8, and EC10), and the three *S. aureus* strains (SA3, SA8, and SA10)] were selected for further investigation using GCBP, RCBP & SCPG extracts. 

**Table 2:** Classification of P. aeruginosa, E. coli and S. aureus for biofilm activity as strong, moderate, and weak (data are n (%) unless otherwise indicated).

Organisms	Strong; n(%)	Moderate; n(%)	Weak; n(%)	Negative; n(%)
P. aeruginosa (n=15)	4(26)	4(26)	5(33)	3(20)
E. coli (n=15)	3(20)	3(20)	4(26)	5(33)
S. aureus (n=15)	3(20)	4(26)	5(33)	3(20)

Correlation analysis 

**Figure 1:**
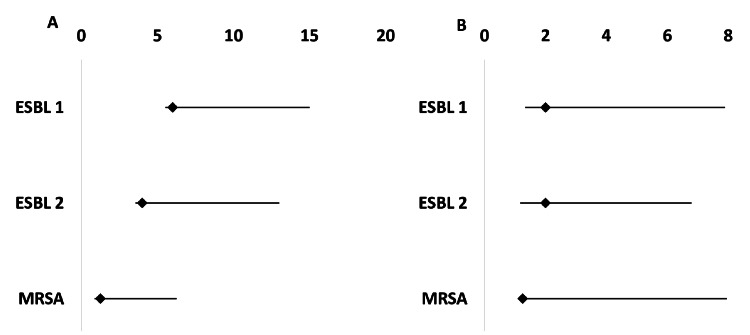
Fox plot regression analysis (A: odds ratio; B: relative risk) at 95% CI of biofilm producing activity in ESBL and MRSA ESBL1: Ceftazidime / ceftazidime clavulanuc acid, ESBL 2: cefotaxime/ cefotaxime clavulanic acid MRSA: MRSA by oxacillin ESBL: extended-spectrum beta-lactamase, MRSA: methicillin-resistant *Staphylococcus aureus*

The study evaluated the probability (odds ratio: OR) and relative risk (risk ratio: RR) of biofilm positivity associated with ESBL and MRSA, comparing biofilm formers and non-biofilm formers (including those with intermediate, weak, or negative biofilm characteristics). Factors exhibiting a positive correlation with biofilm activity were identified as ESBL1 (ceftazidime; OR 6, RR 2), ESBL2 (cefotaxime; OR 4, RR 2), and MRSA (oxacillin; OR 1.25, RR 1.25) (Table 3, Figure 1).

**Table 3:** Logistic regression analysis (OR: odds ratio; RR: relative risk) at 95% CI of biofilm producing activity in ESBL and MRSA ESBL1: ceftazidime / ceftazidime cavulanuc acid, ESBL 2: cefotaxime/ cefotaxime clavulanuc acid, MRSA: MRSA by oxacillin ESBL: extended-spectrum beta-lactamase, MRSA: methicillin-resistant *Staphylococcus aureus*

	OR (95% CI)	RR (95% CI)
ESBL 1	6 (0.47-9.02)	2 (0.83-4.80)
ESBL 2	4 (0.44-0.01)	2 (0.67-5.90)
MRSA	1.25 (0.38-5.21)	1.25 (0.15-9.91)

MICs of GCBP, RCBP, and SCPG extracts

The MICs of GCBP, RCBP, and SCPG extracts were determined against the ESβL-positive, strong biofilm-forming strains of *P. aeruginosa, E. coli, and S. aureus*. The MIC values ranged from 150 to 1000 µg/ml across all tested pathogens. Specifically, the MIC of GCBP exhibited comparable antibacterial activity, with a value of 300 μg/ml noted against two strains of *P. aeruginosa *(PS2, PS11), one strain of *E. coli* (EC1), and one strain of *S. aureus* (SA8). Conversely, certain strains (PS14, EC8, and SA3) displayed higher MIC values of 450 μg/ml for GCBP. Comparatively, the MIC of RCBP indicated higher values than that of GCBP. Notably, among the tested extracts, SCPG extract exhibited the highest MIC values when compared with RCBP and GCBP. The results of the MIC determination are summarized in Tables 4-6. Sublethal concentrations for the biofilm inhibition assay were calculated as 1/2xMIC, 1/5xMIC, and 1/8xMIC. In this study, the high MIC values could be attributed to biofilm-positive and ESBL-positive strains.

**Table 4:** MIC of GCBP extract for strong biofilm and ESBL producing P. aeruginosa, E. coli, and S. aureus strains Sub-MIC were selected for biofilm inhibition assay by GCBP extract.

MIC of GCBP extract (µg/ml)		Sub-MIC of GCBP extract (µg/ml)		
P. aeruginosa		1/8xMIC	1/4xMIC	1/2xMIC
PS2	300	37.5	75.0	150.0
PS3	350	43.7	87.5	175.0
PS11	300	37.5	75.0	150.0
PS14	450	56.2	112.5	225.0
E. coli				
EC1	300	37.5	75.0	150.0
EC8	450	56.2	112.5	225.0
EC10	350	43.7	87.5	175.0
S. aureus				
SA3	450	56.2	112.5	225.0
SA8	300	37.5	75.0	150.0
SA10	350	43.7	87.5	175.0

**Table 5:** MIC of RCBP extract for strong biofilm and ESBL producing P. aeruginosa, E. coli and S. aureus strains Sub-MIC were selected for biofilm inhibition assay by RCBP extract.

MIC of RCBP extract (µg/ml)		Sub-MIC of RCBP extract (µg/ml)		
P. aeruginosa		1/8xMIC	1/4xMIC	1/2xMIC
PS2	350	43.7	87.5	175.0
PS3	400	50.0	100.0	200.0
PS11	200	25.0	50.0	100.0
PS14	500	62.5	125.0	250.0
E. coli				
EC1	350	43.7	87.5	175.0
EC8	500	62.5	125.0	250.0
EC10	400	50.0	100.0	200.0
S. aureus				
SA3	500	62.5	125.0	250.0
SA8	350	43.7	87.5	175.0
SA10	400	50.0	100.0	200.0

**Table 6:** MIC of SCPG extract for strong biofilm and ESBL producing P. aeruginosa, E. coli, and S. aureus strains Sub-MIC were selected for biofilm inhibition assay by SCPG extract. PS: *P. aeruginosa* strain, EC:* E. coli *strain, SA: *S. aureus* strain

MIC of SCPG extract (µg/ml)		Sub-MIC of SCPG extract (µg/ml)		
P. aeruginosa		1/8xMIC	1/4xMIC	1/2xMIC
PS2	550	68.7	137.5	275.0
PS3	650	81.2	162.5	325.0
PS11	450	56.2	112.5	225.0
PS14	700	87.5	175.0	350.0
E. coli				
EC1	550	68.7	137.5	375.0
EC8	700	87.5	175.0	350.0
EC10	650	81.2	162.5	325.0
S. aureus				
SA3	700	87.5	175.0	350.0
SA8	550	68.7	137.5	275.0
SA10	650	81.2	162.5	325.0

Effect of sub-MICs of GCBP, RCBP, and SCPG extract on viability of test bacteria

The introduction of GCBP, RCBP, and SCPG extracts at their respective 1/2x MIC concentrations at the onset of the growth did not lead to any notable alterations in the growth of *P. aeruginosa *strains (PS2, PS3, PS11, and PS14). Similarly, no significant changes in cell densities were observed between treated and non-treated groups in the case of *S. aureus* strains (SA3, SA8, and SA10) and *E. coli* strains (EC 1, EC8, and EC10). Consequently, sub-MICs opted for the investigation of biofilm inhibition to prevent any decline in biofilm growth due to potential growth inhibition.

Inhibition of biofilm by GCBP, RCBP, and SCPG extract in mono and mixed culture

The potential of GCBP, RCBP, and SCPG extracts to hinder biofilm formation at sub-MIC levels (1/2xMIC and 1/16xMIC) was assessed against selected monocultures and mixed cultures. The results of the biofilm inhibition assay are presented in Table 7. Concentration-dependent effects of GCBP, RCBP, and SCPG extracts were observed across all tested strains. GCBP extract exhibited a notable reduction in biofilm formation, averaging 76% for *P. aeruginosa*, 62% for *E. coli*, and 53% for *S. aureus* at 1/2xMIC. RCBP and SCPG extracts demonstrated biofilm inhibition of 66%, 53%, and 51% for P. aeruginosa, E. coli, and S. aureus, respectively. The efficacy of the extract's biofilm inhibition capability against mixed cultures [PS2+SA8, PS3+SA10, PS14+SA3, EC1+SA8, EC10+SA10, EC8+SA3, PS2+EC1, PS3+EC10, PS14+EC8, PS2+EC1+SA8, PS3+EC10+SA10, and PS14+EC8+SA3] was tested and presented in Table 8. GCBP extract demonstrated 66%, 56%, and 17% inhibition at 1/2xMIC, 1/4xMIC, and 1/8xMIC, respectively. RCBP and SCPG extracts exhibited 59%, 33%, 13% and 50%, 28%, and 11% inhibition at 1/2xMIC, 1/4xMIC, and 1/8xMIC, respectively (Table 8). Based on the results from the individual and mixed strain assays, strains PS3, EC10, and SA10 were chosen for further analysis. This concentration-dependent assay supported the selection of GCBP extract for subsequent analyses.

**Table 7:** Reduction percentage of sub-inhibitory concentrations for monoculture at 1/16xMIC-&-1/2xMIC; of GCBP, RCBP and SCPG extract against biofilm formation. All results were in percentage (%) PS: *P. aeruginosa* strain, EC: *E. coli *strain, SA: *S. aureus* strain

Monoculture	GCBP extract		RCBP extract		SCPG extract	
	Reduction percentage		Reduction percentage		Reduction percentage	
	1/16xMIC	1/2xMIC	1/16xMIC	1/2xMIC	1/16xMIC	1/2xMIC
PS2	25%	74%	20%	60%	23%	60%
PS3	30%	80%	24%	65%	21%	63%
PS11	24%	77%	21%	72%	19%	56%
PS14	26%	73%	22%	65%	24%	60%
EC1	24%	69%	16%	59%	19%	51%
EC8	20%	58%	11%	45%	17%	46%
EC10	23%	60%	15%	55%	21%	55%
SA3	21%	55%	13%	53%	11%	50%
SA8	20%	50%	11%	53%	14%	53%
SA10	11%	53%	9%	47%	9%	55%
Average Reduction in PS	26%	76%	22%	66%	22%	60%
Average Reduction in EC	22%	62%	14%	53%	19%	51%
Average Reduction in SA	17%	53%	11%	51%	11%	53%

**Table 8:** Reduction percentage of sub-inhibitory concentrations for mixed culture at 1/16xMIC-1/8xMIC-1/4xMIC-1/2xMIC] of GCBP, RCBP and SCPG extract against biofilm formation. All results were in percentage (%) PS: *P. aeruginosa *strain, EC: *E. coli *strain, SA: *S. aureus* strain

Mixed culture	GCBP extract			RCBP extract			SCPG extract		
	Reduction percentage			Reduction percentage			Reduction percentage		
	1/8xMIC	1/4xMIC	1/2xMIC	1/8xMIC	1/4xMIC	1/2xMIC	1/8xMIC	1/4xMIC	1/2xMIC
PS2+SA8	19%	54%	69%	16%	35%	62%	14%	25%	54%
PS3+SA10	16%	60%	70%	14%	39%	66%	10%	30%	50%
PS14+SA3	20%	65%	71%	17%	40%	64%	11%	32%	45%
EC1+SA8	15%	55%	65%	9%	42%	59%	13%	29%	43%
EC10+SA10	21%	57%	64%	17%	37%	61%	15%	30%	52%
EC8+SA3	15%	60%	65%	11%	35%	60%	6%	27%	50%
PS2+EC1	18%	65%	70%	15%	40%	71%	12%	34%	65%
PS3+EC10	14%	55%	73%	10%	24%	74%	9%	27%	67%
PS14+EC8	16%	60%	67%	12%	35%	65%	17%	25%	60%
PS2+EC1+SA8	18%	50%	60%	11%	24%	39%	9%	27%	35%
PS3+EC10+SA10	15%	48%	57%	10%	28%	40%	8%	21%	40%
PS14+EC8+SA3	19%	40%	55%	14%	22%	45%	11%	24%	41%
Average reduction	17%	56%	66%	13%	33%	59%	11%	28%	50%

Effect on EPS production

**Figure 2:**
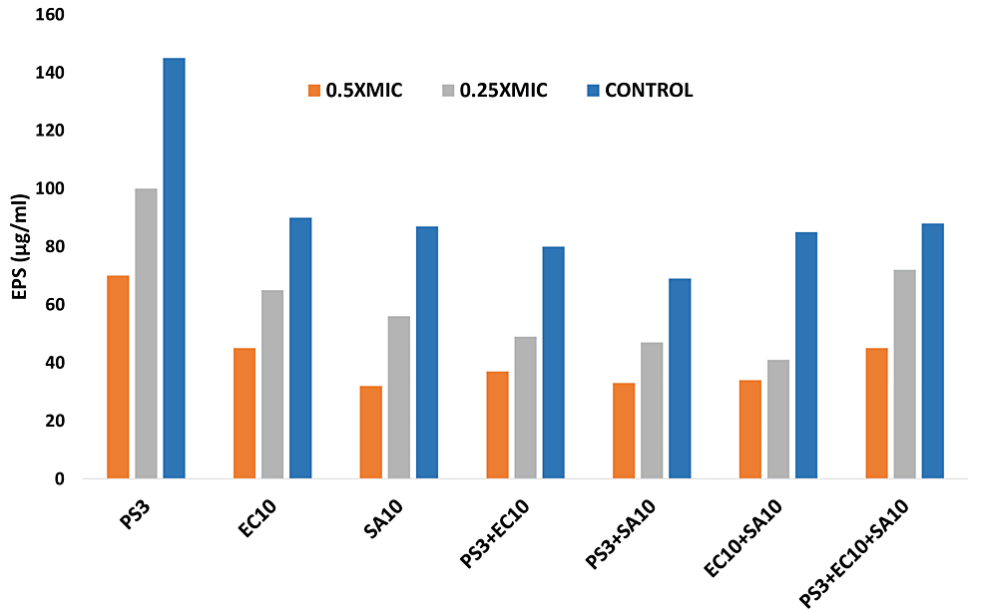
The effect of sub-MIC concentrations on Exopolysaccharides in tested strains. The data are represented as mean values. All tested strains were significantly different (p≤0.05) for all in mono and mixed cultures at 0.25xMIC from untreated control; PS3, EC10, and PS3+EC10+SA10 strains were significantly different (p ≤ 0.005) at 0.5xMIC from untreated control. PS:* P. aeruginosa* strain, EC: *E. coli *strain, SA: *S. aureus* strain

Because of the significant correlation of EPS in the biofilm formation, the test strains were subject to the sub-MIC of GCBP extract to estimate its role in EPS production and non-treated strains showed there was a similar reduction at 1/4xMIC when tested alone, although the inhibition was observer significant reduction in treated strains at 1/2xMIC in all test strains. A similar pattern of inhibition was recorded in mixed strains also (Figure 2). GCBP extracts at respective 1/2xMIC in PS3, EC10, SA10, PS+EC10, PS3+SA10, EC10+SA10 and PS3+EC10+SA10 by 51.7%, 50%, 63.2%, 53.8%, 52.2%, 60%, and 48.9% respectively.

Effect on CSH

**Figure 3:**
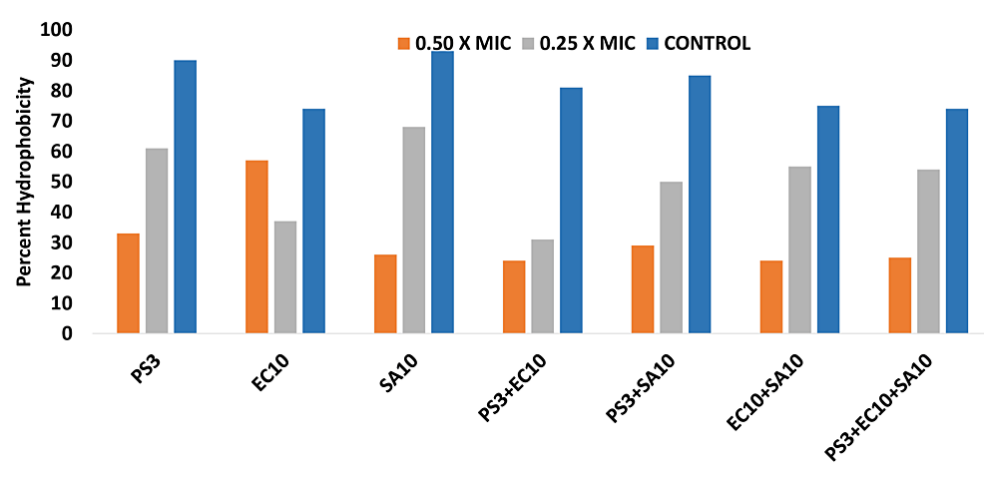
Inhibitory effect of sub-MIC concentrations on CSH in tested strains. The data are represented as mean values. PS3, EC10, and SA10 strains were significantly different (p≤0.05) in mono and mixed cultures at 0.25xMIC from the untreated control. PS3, SA10, and the PS3+SA10 strains were significantly different (p ≤ 0.005) at 0.5xMIC from the untreated control. EC10+SA10 and PS3+SA10+EC10 strains were significantly different (p ≤ 0.001) from untreated control at 0.5xMIC PS: *P. aeruginosa *strain, EC: *E. coli *strain, SA: *S. aureus* strain

The effect on CSH by the GCBP was computed against test bacteria at sub-MIC (Figure 3). It was estimated that CSH was reduced to 33%, 37%, and 26% in mono strains (PS3, EC10, and SA10 respectively) and 24%, 29%, 24%, and 25% in mixed strains (PS3+EC10, PS3+SA10, EC10+SA10, and PS3+EC10+SA10 respectively). CSH observed in untreated control strains was significantly high in monoculture (PS3, EC10, and SA10) as well as in mixed cultures (PS3+EC10, PS3+SA10, EC10+SA10, and PS3+EC10+SA10).

Effect on the outer membrane disruption

**Figure 4:**
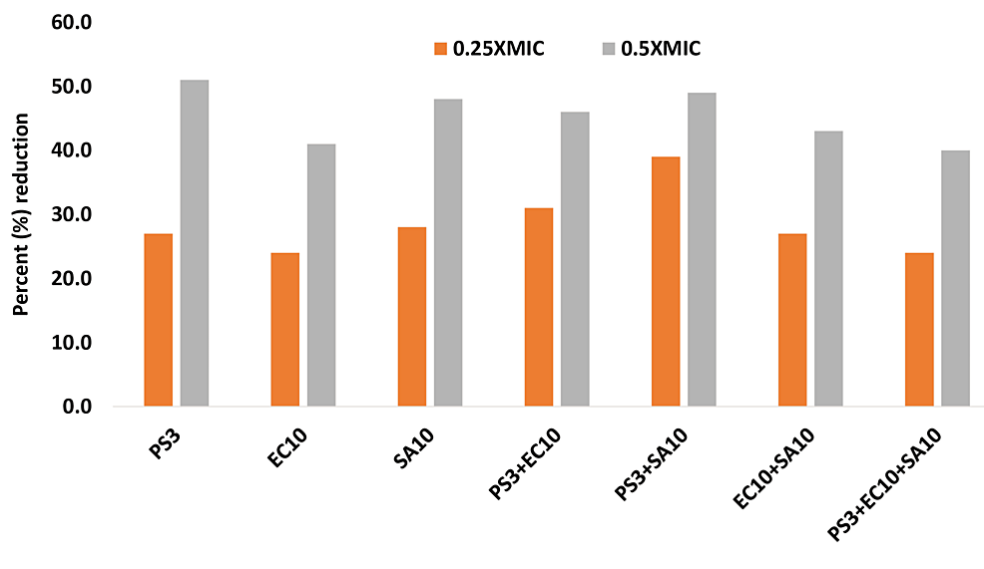
Influence of sub-MIC concentrations on outer membrane disruption in tested strains Data are represented as mean values. PS3, EC10, and PS3+EC10 strains were significantly different (p≤0.05) at 0.25xMIC from the untreated control. PS3, EC10, PS3+EC10, E10+SA10, and PS3+EC10+SA10 strains were significantly different (p ≤ 0.005) at 0.5xMIC from the untreated control. PS3+SA10 strains were significantly different (p ≤ 0.001) from the untreated control at 0.5xMIC. PS: *P. aeruginosa* strain, EC: *E. coli *strain, SA: *S. aureus* strain

The potent role of coffee extract on bacteria (mono and mixed) was determined using an N-Phenyl-1-naphthylamine (NPN) uptake assay to assess outer membrane disruption. NPN cannot normally be inserted into bacterial membranes; however, when GCBP extract disrupts the outer membrane, NPN penetrates the lipid layers, increasing the intensity of its fluorescence emission. It was estimated that NPN uptake was increased to 51%, 41%, and 48% in mono strains (PS3, EC10, and SA10 respectively) and 46%, 49%, 43%, and 40% in mixed strains (PS3+EC10, PS3+SA10, EC10+SA10, and PS3+EC10+SA10 respectively) (Figure 4). The NPN at sub-MIC (1/4xMIC) also demonstrated a similar pattern but the uptake compared to 1/2xMIC is low. 

Effect on motility

**Figure 5:**
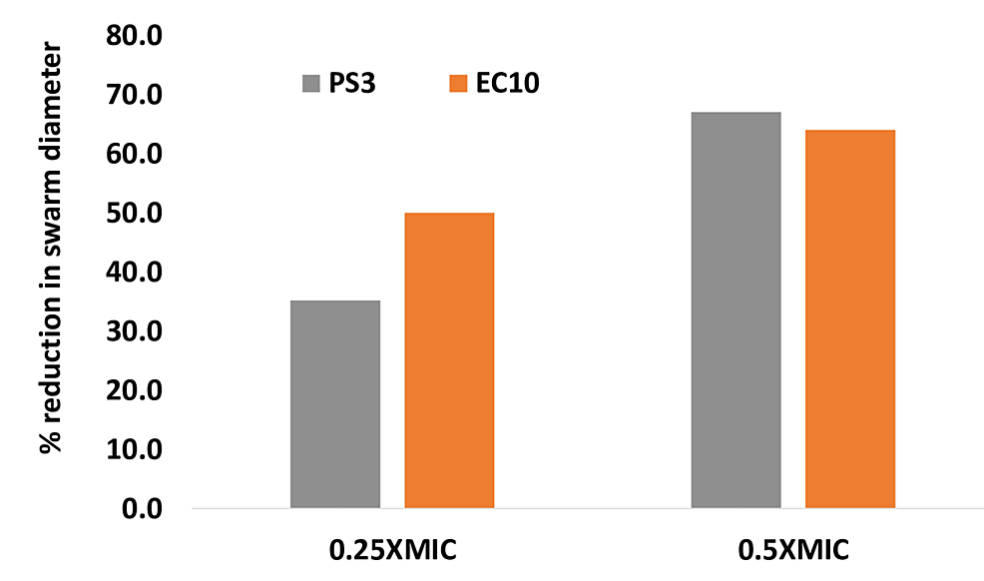
Effect on swarming motility of sub-MIC concentrations in tested strains. The data are represented as mean values. PS3 strains were significantly different (p≤0.05) at 0.25xMIC from the untreated control. EC10 (0.25xMIC and 0.5MIC) strains were significantly different (p ≤ 0.005) from the untreated control. PS3 strains were significantly different (p ≤ 0.001) from the untreated control at 0.5xMIC. PS: *P. aeruginosa *strain, EC: *E. coli* strain, SA: *S. aureus* strain

The role of flagella in motility is vital for the survival, proliferation, and pathogenicity of *E. coli *and *P. aeruginosa*. Impressively, the motility was significantly reduced in all the tested strains (at respective sub-MIC) compared with untreated (Figure 5). The size of the zone of inhibition of the swarm corresponded with the concentration of GCBP extract 1/4xMIC to 1/2xMIC. The highest inhibition of zone at 1/2xMIC of 67% and 64%. This increase in the inhibition indicates the activity of GCBP in inhibiting the formation of biofilm. 

Effect on chitinase activity

**Figure 6:**
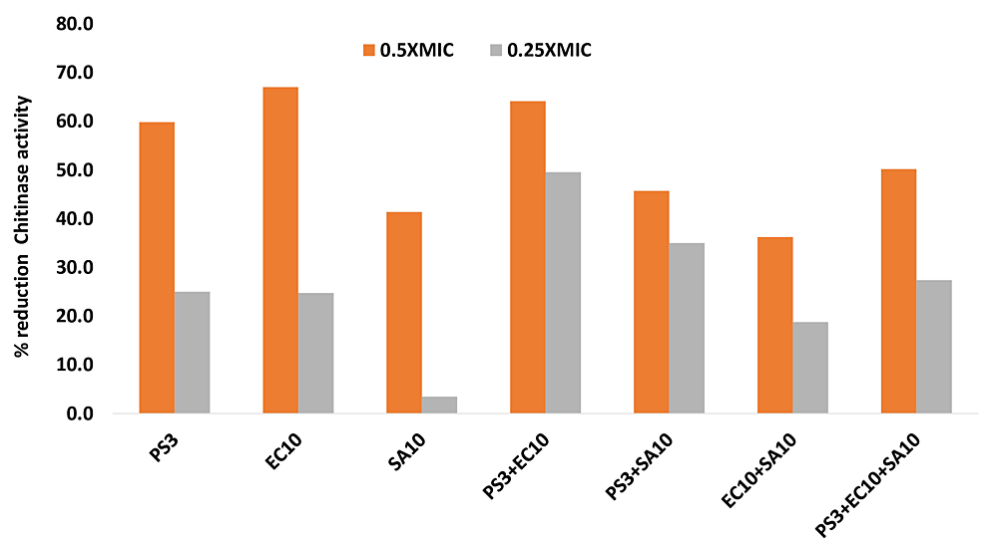
Effect on chitinase activity of sub-MIC concentrations in tested strains. The data are represented as mean values. PS3, EC10, SA10, PS3+EC10, PS3+SA10, and PS3+EC10+SA10 strains are significantly different (p≤0.05) at 0.25xMIC from the  untreated control. PS3, EC10, PS3+EC10, PS3+SA10, and PS3+EC10+SA10 strains significantly different (p ≤ 0.005) from the untreated control at 0.5MIC. SA10 and EC10+S10 strains were significantly different (p ≤ 0.001) from the untreated control at 0.5xMIC. PS: *P. aeruginosa* strain, EC: *E. coli* strain, SA: *S. aureus* strain

Chitinase activity in biofilm formation is crucial for comprehending the dynamics of microbial communities in various environments. It was estimated that chitinase activity was reduced to 59.8%, 67%, and 41.4% at 1/2xMIC when estimated in monoculture (PS3, EC10, and SA10) compared with untreated control strains in monoculture and a similar reduction was observed in chitinase activity in mixed strains (PS3+EC10, PS3+SA10, EC10+SA10, and PS3+EC10+SA10 respectively) at 1/2xMIC. Similar results were also depicted at 1/4xMIC for mono and mixed cultures (Figure 6). 

 Effect on pre-formed biofilm

**Figure 7:**
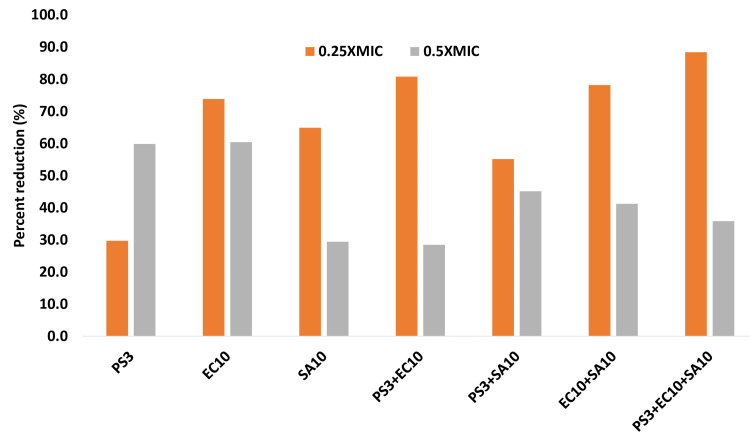
Effect of sub-MIC on disruption of pre-formed biofilm of sub-MIC concentrations in tested strains. The data are represented as mean values. PS3, EC10, PS3+EC10 strains significantly different (p≤0.05) at 0.25xMIC from untreated control. PS3, EC10, PS3+EC10, E10+SA10, and PS3+EC10+SA10 strains were significantly different (p ≤ 0.005) at 0.5xMIC from untreated control. PS3+SA10 strains were significantly different (p ≤ 0.001) from untreated control at 0.5xMIC. PS:* P. aeruginosa* strain, EC: *E. coli *strain, SA: *S. aureus *strain

The disruption of preformed biofilms is significant for several reasons; it is primarily associated with public health, industrial processes, and environmental considerations. The presence of biofilms on medical devices, implants, and tissues can lead to persistent infections that are difficult to treat with conventional antibiotics. It is also very difficult to disrupt the pre-formed biofilm by increasing the antibiotic concentration which poses a hidden threat to diabetic patients. The GCBP extract potential to disrupt the preform 24 hr old biofilm at 1/2x and 1/4x MIC against all test strains of ESBL and MRSA and in combination with MRSA+ESBL. Figure 6 demonstrated that at the sub-MIC, there was significant disruption of preform biofilm compared with untreated controls. In monocultures, the reduction was recorded as 59% and 60.4% in ESBLs (PS3 and EC10) and 29.4% in MRSA [SA10]. In the case of mixed cultures (MRSA+ESBLs), the highest reduction was observed ass 45.5% by PA3+SA10 followed by 41.2% by EC10+SA10 at 1/2XMIC; whereas only there was only a 28.4% reduction in a mixed culture of ESBLs (PS3+EC10() (Figure 7).

## Discussion

The widespread consumption of coffee especially in the Middle East region highlights its position as one of the most enjoyed beverages worldwide. It then emphasizes the potential benefits of integrating natural products into our daily diets for disease prevention, suggesting that this approach might offer enhanced safety and efficacy, as referenced by a specific source [1]. This study elaborates on the anti-bacterial and anti-biofilm properties of extracts derived from coffee beans and coffee by-products. Among these extracts, GCBP exhibited the most promising efficacy at MIC and sub-MIN levels; it also exhibited anti-biofilm activity in mono and mixed culture when used for in vitro experiments against the three most common bacteria seen in DFUs: *P. aeruginosa, E. coli, and S. aureus*. By exploring the antibacterial and antibiofilm activities of coffee extracts against these pathogenic bacteria, the study aims to determine whether compounds within the coffee extract could inhibit the growth or formation of bacterial biofilms at its MIC and sub-MIC levels and in mono and mixed culture in-vitro analysis.

Understanding these potential effects could have implications for infection control and might provide insights into novel methods for preventing or managing DFUs caused by these specific bacteria. This research could contribute to the development of alternative strategies or treatments in DFUs. Antibiotics are experiencing a decline in effectiveness owing to the rising prevalence of bacterial antimicrobial resistance. As highlighted in a WHO report, antimicrobial resistance stands among the top 10 global public health concerns due to the inappropriate usage and excessive application of medications, encompassing anti-bacterial drugs [23]. Consequently, there's a pressing need for alternative therapeutic methods. In the context of diabetic foot complications, biofilm formation by bacterial strains such as *P. aeruginosa, E. coli, and S. aureus* is of particular concern. DFUs are a common complication of diabetes that can become infected and often develop into chronic wounds. A high biofilm positivity rate indicates that a substantial portion of diabetic foot ulcers are affected by bacterial biofilms. This is a matter of concern as biofilms present a significant challenge in the management of these wounds due to their resistance to conventional antibiotics and immune responses, leading to prolonged healing times and increased risk of complications [8]. The investigation into the antimicrobial properties of GCBP, RCBP, and SCPG extracts has become increasingly significant due to the rise in bacterial resistance to conventional medications [9]. A few studies also reported similar results of MIC but from non-Middle East regions against *E. coli, S aureus, Listeria monocytogenes, and Enterococcus faecalis* [9-12]. The results of this study demonstrate that the green coffee bean extract shows promising results and demonstrated a significant antibiofilm activity against monoculture (1/16 MIC and ½ MIC) and mixed culture (1/8MIC, 1/4MIC and 1/2MIC) in its sum-MIC concentrations. Notably, RCBP and SCPG extracts did not display significant antibiofilm activity compared with GCBP against the tested bacterial strains at its sub-MIC level.

The extracellular polymeric substances (EPS) within biofilms serve as the fundamental scaffolding for microbial communities, orchestrating pivotal functions crucial for their survival and persistence. Initially, EPS facilitates the irreversible attachment of microbial cells to surfaces, initiating biofilm formation. This matrix acts as a stronghold, providing structural support, thereby maintaining the biofilm's stability and resilience against external disturbances. Beyond mere structural integrity, EPS serves as a protective shield, safeguarding embedded cells from harsh environmental conditions and hindering the entry of antimicrobials, including antibiotics, while deflecting assaults from the host immune system. Furthermore, EPS houses specialized enzymes essential for the degradation of external substances, aiding in nutrient cycling and maintaining the biofilm's metabolic processes. In essence, EPS orchestrates a multifaceted defense and sustenance mechanism, enabling biofilms to thrive, adapt, and persist in diverse and often hostile environments [24]. Study observations pertaining to the inhibition of EPS indicate that a reduction in EPS production could render cells susceptible to the impact of the extract, potentially leading to the elimination of the biofilm. Additionally, the exposed sessile cells are anticipated to exhibit heightened susceptibility to antibacterial agents and human leukocytes. To our knowledge, there have been no prior documented instances of the GCBP extract impairing exopolysaccharide inhibition in the tested strains (*P. aeruginosa, E. coli, and S. aureus*).

The hydrophobicity index of bacteria, representing their tendency to repel water, holds substantial importance in the initial stages of biofilm formation. Consequently, targeting and reducing the cell surface hydrophobicity (CSH) is considered an effective approach to impede the formation of biofilms. The results obtained from the MATH assay provide clear evidence that bacteria treated with GCBP extract exhibited a significant decrease in cell surface hydrophobicity compared to untreated bacteria. This reduction following GCBP treatment suggests its potential to alter the surface characteristics of bacterial cells, making them less prone to adhere and initiate biofilm formation. This study also demonstrates GCBP activity not only with mono-cultures but also with mixed cultures. As per the literature review, this hydrophobicity activity of GCBP is probably the first report against mono and mixed cultures. The disruption of the outer membrane by coffee extract has shown potential in reducing the adherence and attachment of these bacteria to surfaces, which are crucial initial steps in biofilm formation. This study demonstrated the disruption of the outer of *P. aeruginosa, S. aureus, and E. coli* by reducing the biofilm formation capabilities not only in mono-cultures but also in mixed cultures. The plausible reason may be because of the various bioactive compounds such as polyphenols, caffeine, and other secondary metabolites in coffee extract, that have exhibited the ability to interact with bacterial outer membrane. This interaction may lead to alterations in membrane integrity, disruption of protein structures, and interference with essential cellular processes mediated by these proteins [16, 25]. As per the literature survey, the disruption of the outer membrane by GCBP appears to be the first reported instance against both mono and mixed cultures). While these findings are promising, further research is necessary to comprehensively understand the mechanisms by which coffee extract interacts with the outer membrane of these bacteria and inhibits biofilm formation.

Swarming motility, leading the initial attachment of bacterial cells to surfaces, significantly influences biofilm formation. Additionally, it serves as a crucial factor in bacterial virulence, impacting their pathogenicity [16, 26]. The synthesized GCBP extract notably decreased the motility of the tested bacterial strains. This observation suggests a potential application in combating persistent infections caused by drug-resistant *P. aeruginosa and E. coli.* As per the literature search, there have been no prior reports documenting the GCBP extract's ability to impede the motility of *P. aeruginosa* and *E. coli*. Therefore, this discovery represents a significant advancement, potentially introducing a novel approach utilizing GCBP extract to target and restrict the motility of these bacterial strains in the battle against biofilm infections.

In bacteria, the regulatory mechanisms governing the expression of exoproteins utilizing various secretion pathways, such as elastase and alkaline protease, are under the control of a shared quorum-sensing system. While the expression of chitinase also relies on this quorum-sensing system. Elucidating the biological role of chitin is imperative to comprehend the function of chitinase in *P. aeruginosa*, which likely differs from that of chitinase in *Serratia marcescens*. *S. marcescens* efficiently degrades colloidal chitin and utilizes it as the primary carbon source by secreting multiple chitinases and a chitin-binding protein into the extracellular environment [27]. Conversely, the chitinase produced by clinical isolates of *P. aeruginosa* suggests a potential role in pathogenicity. This observation implies that these proteins might contribute to the bacterium's ability to cause infection [28]. With the propensity of tested strains to infect wounds, it is plausible that chitinase may impede wound healing, facilitating bacterial establishment of infection [29]. This study reported the inhibitory effect of GCBP against chitinase activity, not only in mono-culture but also in mixed-culture in-vitro analysis. This inhibition ultimately affected the potency of biofilm formation. The research conducted by Akhlaghi et al. [1] revealed a reduction in bacterial biofilm infections. However, there are no previous studies reporting the reduction of chitin's activity through GCBP extract in diminishing biofilm growth in both monoculture and mixed culture settings. Hence, this finding signifies a significant breakthrough, potentially introducing a new strategy using GCBP extract to mitigate chitinase activity in these bacterial strains, effectively combating biofilm infections.

Several research studies have highlighted the potential of coffee extract in inhibiting and disrupting biofilm formation. These extracts have demonstrated the ability to penetrate and disrupt the matrix of pre-existing biofilms formed by different bacterial species [30]. The disruption of pre-existing biofilms by coffee extracts involves several mechanisms, including interference with quorum sensing, inhibition of bacterial adhesion, and disruption of intercellular communication pivotal for biofilm sustenance. Additionally, the presence of bioactive compounds within coffee extracts contributes significantly to their antimicrobial and antibiofilm properties [25]. It's crucial to recognize that the efficacy of coffee extract against biofilms may vary depending on factors such as the specific bacterial species, the composition of the biofilm matrix, and the concentration or formulation of the extract used in the study. The data presented unequivocally indicate the potential of GCBP (or coffee) extract in eliminating pre-existing biofilms in both single-bacterial and mixed-bacterial infections. The study demonstrates that the coffee extract has the capability to penetrate the biofilm matrix and eliminate approximately 45% of established biofilms formed by all tested bacterial strains. However, while these findings are promising, it is essential to conduct further in vivo studies to mimic actual infections, as in vivo biofilms often exhibit characteristics distinct from most in vitro biofilms [8].

Limitation of the Study

The results obtained in in-vitro showcasing the potential antibacterial and antibiofilm properties of coffee extracts might not entirely reflect their effectiveness within the in-vivo. Real-life scenarios involve complex interactions influenced by factors like diverse biofilm compositions, individual host characteristics, and environmental variables. Hence animal based experimental studies is warranted also.

## Conclusions

The study investigates GCBP's potential as an antimicrobial and antibiofilm agent against prevalent isolates found in DFUs, illustrating its effectiveness in inhibiting biofilm formation in both mono and mixed bacterial cultures. GCBP extract diminishes the production of critical substances that maintain biofilm stability, rendering the biofilm more susceptible to treatment. Furthermore, it influences bacterial attachment and disrupts their outer membranes, impeding biofilm formation. Additionally, the study highlights GCBP's impact on chitinase activity within tested strains, potentially affecting infection and wound healing processes. The inhibition of chitinase activity using GCBP extract signifies progress in addressing biofilm-related infections. This research underscores GCBP's interference with bacterial biofilm formation, cell attachment, substance production, and outer membrane integrity. Overall, this study suggests a breakthrough in utilizing GCBP as an alternative strategy to control bacterial growth and biofilm formation, especially in the context of managing diabetic foot ulcers, offering an alternative to conventional antibiotics. In essence, the future holds promise for GCBP as a potential therapeutic agent against biofilm-related infections in DFUs. However, further comprehensive research, clinical trials, and optimization efforts are necessary to fully realize its potential in clinical applications.
